# Effect of manual therapy on music students with playing-related musculoskeletal disorders: a prospective study

**DOI:** 10.3389/fpain.2023.1151886

**Published:** 2023-06-28

**Authors:** Carolin Assel, Boya Nugraha, Nicolas Kallusky, Stephan Faßnacht-Lenz, Eckart Altenmüller, Christoph Gutenbrunner, Christian Sturm

**Affiliations:** ^1^ Department of Rehabilitation Medicine, Hanover Medical School, Hanover, Germany; ^2^ Institute of Music Physiology and Musicians’ Medicine, Hanover University of Music, Drama and Media, Hanover, Germany; ^3^ Physiotherapy Practice, Faßnacht-Lenz Stephan, Hanover, Germany

**Keywords:** playing-related musculoskeletal disorder, manual therapy, hypermobility, music students, musician, pain, music

## Abstract

Playing Related Musculoskeletal Disorders (PRMD) belong to the most prevalent medical ailments affecting musicians' health and career. This study documents the effect of a physiotherapeutic treatment as well as functional impairments of PRMD on the musculoskeletal system. In total, 32 music students suffering from PRMD were examined in Hanover Medical School (MHH) before and after they received twelve physiotherapeutic treatments, which were carried out over 20 min each over 6 weeks. Additionally, 32 healthy music students, matched by age and gender, were examined at one time point in the MHH to explore which musculoskeletal restrictions are associated with PRMD. The examination included the evaluation of the pain on the Visual Analogue Scale for pain (VAS), a body composition, and body posture measurement, the finger-to-floor distance, the range of motion of the cervical spine, the pressure pain and muscular hypertension examination, the temporomandibular joint-test, the Beighton score screening test, and the testing of the widespread pain score (WSP). After analyzing the data of the patient group (PG) a significant reduction of pain level on the VAS from an average pain of 5.33 to 3.35 was found (large effect). Additionally, a significant reduction of the pressure pain of the M. levator scapulae, the M. rhomboideus, the M. sternocleidomastoideus on the left side and the paravertebral muscles of the cervical spine on the right side after the treatment of the patients could be detected. Regarding the WSP, the positive testing significantly differed, showing a 28% positive testing in the patient group vs. a positive testing of 9% in the control group (CG). As hypermobility is a common phenomenon in musicians, the percentage of those being diagnosed with generalized hypermobility by using the Beighton score in both groups (PG: 37.5%; CG: 25%) was remarkably higher compared to previous studies. In this study, a short course of manual therapy, client tailored for each musician's specific problem, was shown to reduce pain levels in musicians with PRMD.

## Introduction

When experienced by non-musicians, listening to music frequently arouses emotions and physical reactions of wellbeing and relaxation. In contrast, when a person plays an instrument professionally, the activity can often lead to discomfort and painful muscle maladjustments, such as Playing Related Musculoskeletal Disorder (PRMD). The lifetime prevalence of musculoskeletal complaints in professional musicians ranges from between 62% and 93% and therefore represents a huge health restriction for musicians (1–5). However, professionals are not the only musicians affected: a study investigating the prevalence of PRMD among music students in Europe found that 48% of participants were affected by self-reported PRMD (6). Steinmetz and colleagues reported that 81% of 36 music students suffered from pain and discomfort while playing their instrument (7). Out of 330 freshmen music students, 79% reported a history of PRMD (8). In another study of classical piano students in Malaysia, 35.8% of the students reported having PRMD (9).

Regarding the percentage of musicians affected by PRMD, it should be mentioned that the definition of PRMD is not consistently used. Zaza, Charles, and Muszynski provide a well-established description of PRMD as “pain, weakness, lack of control, numbness, tingling, or other symptoms that interfere with your ability to play your instrument at the level you are accustomed to” (10). Since musculoskeletal pain affects more musicians than non-musicians (11), some risk factors for PRMD have been described. These include a high number of practicing hours (12), as well as an immediate increase in playing time (13). Inherited musculoskeletal characteristics and playing techniques also seem to influence the musculoskeletal well-being of musicians (14). Furthermore, the musculoskeletal symptoms differ according to the instrument played. In a survey of 441 musicians from six Danish symphony orchestras, woodwind players had a lower risk in comparison with other instrumentalists and there was a higher prevalence of musculoskeletal symptoms in women than in men (15). Upper string players also seem to have a higher risk of suffering from musculoskeletal disorders (16, 17).

Even though PRMD is very common among musicians, to date, little research has been conducted to investigate whether preventive strategies and therapy for PRMD exist. Zaza examined 281 classically trained professionals and university student musicians. They identified warming up before and taking breaks during practice sessions as preventive factors of PRMD (18). Additionally, it is recommended, that the treatment of musicians should include education and advice, specialized onsite injury and recovery services, cross-training exercise regimes, music performance biomechanic feedback, and ergonomic considerations (19). It has also been shown that a psychosocial course tailored to music students (PRESTO-Play) and a course providing education about physical activity recommendations for the general population (PRESTO-Fit) could reduce the percentage of music students with performance related disability (20). Similarly, manual therapy improved movement parameters such as range of joint mobility, force measures, and coordination among musicians patients with pain disorders (21). Even though the positive impact of physical therapy on musculoskeletal disorders in musicians is acknowledged (22), studies proving the effectiveness of physiotherapeutic interventions related to PRMD are rare.

Since the majority of previous studies investigating musculoskeletal dysfunctions in musicians have used questionnaires (3, 23), this study was established to explore musculoskeletal restrictions correlating with PRMD using a detailed physical examination. As PRMD can be a severe health restriction and may even force musicians to end their careers, knowledge about effective therapy concepts can help to decrease the number of musicians suffering from PRMD.

This study aimed to explore musculoskeletal restrictions correlating with PRMD by using a detailed physical examination. The results were not only compared before and after a 12-session physiotherapeutic treatment but also a healthy control group of music students not suffering from PRMD. Specifically, this study aimed to explore the effects of Manual Therapy by comparing bodily exams before and after a physiotherapeutic treatment.

## Methods

### Study design

#### Section A

The study is a prospective cohort study with an uncontrolled trial and an effective experimental design. It was conducted at the Hanover Medical School (MHH) in cooperation with the Institute of Music Physiology and Musicians' Medicine (IMMM) of the Hanover University of Music, Drama, and Media. It is part of a cooperation study divided into psychological factors influencing the development of PRMD (IMMM), as part of which this study focuses on the somatic factors that determine the origin of PRMD. Data on the psychological impact of PRMD will be published elsewhere (24).

In total, 32 music students (23 female, 9 male) suffering from PRMD (patient group, PG) were physically examined before a closely spaced twelve session physiotherapeutic treatment (timepoint T1), which included a 20 min treatment per session specific to the participants' main symptoms carried out by two expert physiotherapists from a physiotherapy clinic that specializes in musicians and directed by co-author Stephan Faßnacht-Lenz. The participants were asked to finish the treatment within 6 weeks.

The detailed examination included the evaluation of pain on the Visual Analogue Scale (VAS), a body composition- and body posture measurement, the finger to floor distance, the range of motion of the cervical spine, pressure pain and muscular hypertension examination, the temporomandibular joint-test, the Beighton score screening test, and a Widespread Pain Score (WSP) was calculated.

The same parameters and examinations were collected after the therapy (timepoint T2) to show the effectiveness of the physiotherapeutic treatment.

#### Section B

This study aimed to answer the question, and was concerned with the musculoskeletal restrictions of PRMD and predisposing factors such as hypermobility linked to the development of PRMD. Therefore the control group (CG), which consisted of music students not suffering from PRMD, underwent a physical examination identical to the patients' group.

All subjects provided written informed consent and the study protocol received ethics approval from the ethics committee at Hanover Medical School (MHH) (study number 2865-2015). The study was conducted in accordance with the principles of the Declaration of Helsinki.

### Participants

Participants were recruited by the IMMM, either by being asked during a PRMD appointment in the musician's clinic or by a call asking them to participate in the study based on patient records indicating that they suffered from PRMD. We also placed notices inside the Hanover University of Music, Drama and Media building. After a screening examination to verify the diagnosis of PMRD, an appointment at the MHH was made for a detailed bodily examination.

The healthy subjects in the control group were also recruited in the IMMM and, after we had ascertained that they did not have PRMD (based on the previously outlined criteria), they were sent to the MHH to be examined the same way as the patient group (cf. Table 1). The patients and the control group were matched by age, gender, and instrument.

**Table 1:** Demographic data.

	Patients	Healthy Control
Gender *(female/male)*	23/9	23/9
Age *(years)*^a^	22,78	22,86
Instrument		
Strings	12	12
Wind instrument	9	7
Keyboard instrument	6	10
Percussion instrument	2	2
Others/not known	3	1

^a^ All variables are median values.

The following parameters were set up as inclusion and exclusion criteria for the patient group:

➢ Inclusion criteria:
  – suffering from PRMD (diagnostic criteria for PRMD: pain that has arisen in the context of playing the instrument and affects a body part crucial for playing the instrument; it should not be linked to structural damage, be it nerve compression or obvious tissue damage, such as swelling or inflammation of a joint.)
  – aged 18 to 30 years old
  – a pain intensity of three or more on the Visual Analogue Scale (VAS) during the last week
  – not previously exposed to physiotherapeutic treatment for PRMD
➢ Exclusion criteria:

Diseases like cancer, heart failure, major depressive disorder, acute and inflammatory diseases, injury, diabetes, autoimmune and infection diseases, joint and spine diseases, as well as pregnant and breast-feeding females, or only temporary pain (<3 weeks).

The inclusion and exclusion criteria stayed the same for the control group with the exception that the control group participants must be free from relevant pain.

### Clinical examination

To explore musculoskeletal restrictions correlating with PRMD, a detailed physical examination was performed by two experienced physicians at the MHH. For the examination, 45 min were scheduled per patient, and individual extra time was given for filling in the questionnaire. First, the probands had to mark their estimated pain in two separate visual analogue scales (VAS). The first scale represented the average pain felt during the last week (VAS1), and the second one requested the average pain felt after playing their instrument during the last week (VAS2).

Participant’s body composition (height, bodyweight, muscle mass, fatty tissue, and Body-Mass-Index, BMI) was measured using the InBody machine (InBody 230; Model MW160, Korea).

To be able to detect any physical anomalies that might be related to PRMD, the following examinations and tests were carried out:

– Body posture examination, which included the examination of the symmetry of the shoulders (shoulder elevation measured in cm), the detection of spine scoliosis via visual diagnosis and palpation of the spine, the assessment of the waist triangle (visual diagnosis), and the examination of the pelvic position (palpation of the Spina iliaca anterior and posterior superior, deviation measured in cm).
– Finger-floor-distance (25), the distance between the finger and the floor in a bent forward position with stretched knee was measured (for being able to touch the floor with the palmar side of the fingers −5 cm was defined, the bent palmar side of the hand was defined as −10 cm).
– Range of motion of the cervical spine, measured using CROM 3 (26): with a magnetic compass included in a helmet-like head piece the exact range of motion of the cervical spine in inclination, reclination, and the lateral side-bending to the left, as well as to the right side was measured in degree.
– Pressure pain and increased muscle tension of the hyoid muscles, the M. sternocleidomastoideus, M. scaleni, M. trapezius, M. levator scapulae, M. rhomboideus, M. pectoralis, paravertebral muscles of the cervical-, thoracic-, and the lumbar spine and the M. quadratus lumborum were examined by manual palpation carried out by one of the experienced physicians. The depth of the manual palpation was initially calibrated with a Tissue Tensiometer so that the examination was comparable. The variables are dichotomous (where 0 signifies no pressure pain/normal muscle tension, and 1 signifies pressure pain/increased muscle tension). Increased muscle tension was determined by the examiner when an increased resistance of the muscles was palpable. A positive tenderness was recorded as soon as a patient expressed pain on palpation.
– temporomandibular joint test (developed by a study group of dentists of the MHH for detection of temporomandibular disorders) (cf Table 2).
– Beighton score screening test of hypermobility (27). A summation of the Beighton score of 0–2 points was defined as no hypermobility, 3–4 points as moderate hypermobility, and 5 or more points detected manifest hypermobility (28) (cf Table 3, showing the components of the test).
– Testing of the 18 tender points for assessing widespread pain (WSP) (according to testing of WSP in fibromyalgia). A score of 12 or more positive tender points led to a positive WSP testing.

**Table 2:** Temporomandibular joint test.

	Yes	No
Painful palpation of the joint	** **	** **
Joint crepitations? Noises?	** **	** **
Painful palpation of the temporomandibular muscles	** **	** **
Asymmetric mouth opening	** **	** **
Restriction of the mouth opening at the moment	** **	** **
Restriction of the mouth opening ever before	** **	** **
Jaw opening (active) in cm	** **	** **
Jaw opening (passive) in cm	** **	** **

**Table 3:** Beighton score screening test.

The ability to	Right	Left
Place hands flat on the floor without bending the knees	1	
Hyperextend the elbow to ≥10°	1	1
Hyperextend the knee to ≥10°	1	1
Oppose the thumb to the volar aspect of the ipsilateral forearm	1	1
Passively dorsiflex the fifth metacarpophalangeal joint ≥90°	1	1
Total possible score	9	

### Physiotherapy (intervention)

Twelve sessions of physiotherapy were prescribed per patient, carried out by two experienced physiotherapists specializing in the treatment of musicians. The treatment was adapted to the individual symptoms of the musicians, dependent on the results of the physical examination. The therapy included a postural stabilization at the beginning of the treatment, followed by individual treatments (e.g., mobilization, myofascial techniques, core stabilization, therapeutic exercises as sequences of movements and stretches, muscular relaxation techniques, and awareness training for the patients to perform at home). To identify the specific risk factors induced by postural workload or lack of mobility patients were asked to bring their musical instrument. Thereby, therapists could better adapt exercises to patients' problems. After the treatment was completed, a treatment report for every patient was created by the physiotherapists (24).

### Statistical analysis

The study was a sub-study as part of a main study exploring the correlation between BDNF (brain-derived neurotrophic factor) and PRMD. The negative outcome of BDNF analysis (no correlation with pain or therapy) will be reported elsewhere. Additional to these studies, the IMMM examined in another sub-study the psychological factors that influence PRMD. According to the main study the sample size was calculated based on a study by Laske et al. (29). Based on this calculation, 27 patients and 27 healthy subjects would be needed to reject the null hypothesis that the population means of the patients and healthy subjects group were equal with probability (power) 0.9. However, considering that there was an approximate 10% dropout after treatment for secondary endpoint analysis, in the end, at least 30 patients were recruited. The type I error probability associated with this test of this null hypothesis was 0.05.

The statistical analysis of the body examination (T1 and T2) was evaluated by STATA Version 16 using Shapiro-Wilk-Test for detecting a normal distribution, so analysis continued with ANOVA or Kruskal-Wallies-Test chi-squared, or chi-squared with ties. Significance is set at *p* < 0.05.

## Results

### Recruitment

**Figure 1:**
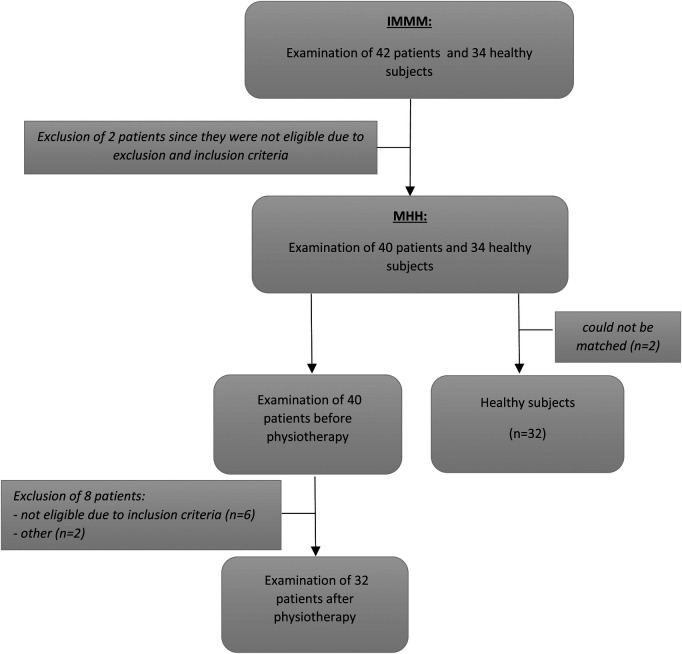
Recruitment of study participants.

From the initial 42 examined patients, ten had to be excluded. Two participants were not eligible due to the inclusion and exclusion criteria, six of the participants did not reach the requested VAS, one had to be excluded since the data collection could not be finished due to the patient's schedule, and one had to be excluded since the matched healthy control participant did not fulfill the criteria of being free from pain. Furthermore, two of the healthy subjects were excluded after going through the examination, since they could not be matched (cf. Figure 1).

### Body measurement

**Figure 2:**
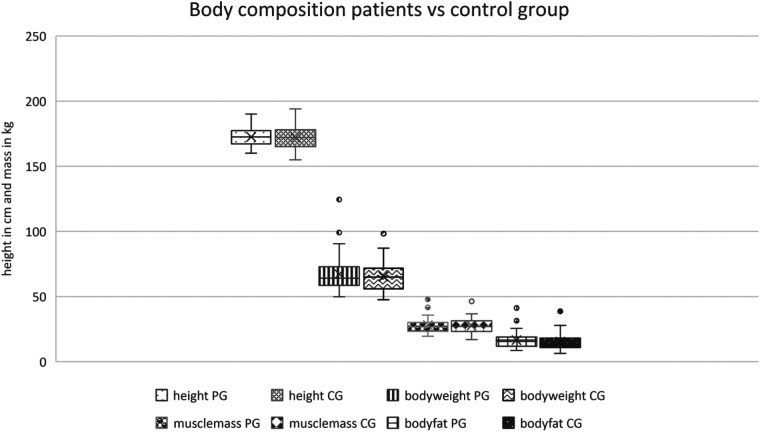
Body composition of patient group vs. control group.

**Figure 3:**
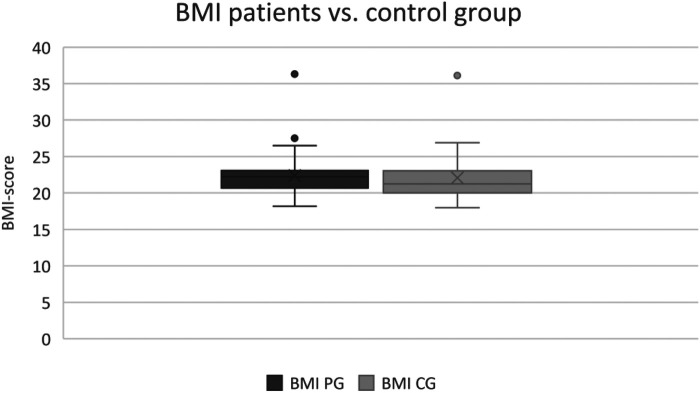
BMI of patient group vs. control group.

The body measurement represented a good match within the control and the patient group, as there was no significant difference in the captured parameters including height, bodyweight, muscle mass, the fatty tissue of the body in kg, as well as the BMI (cf. Figures 2, 3). The body measurement parameters of patients in T1 did not differ compared to the second time point T2, on average.

### Patients vs. healthy control group

**Figure 4:**
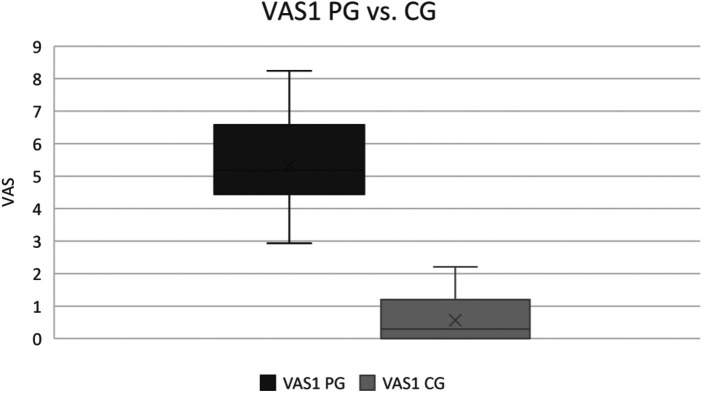
VAS1 of the patient group vs. VAS1 of the control group.

Consistent with our classification of students as healthy subjects or patients, patients' average (maximum) VAS1 was 5.33 (8.24) compared with the average VAS1 for healthy subjects of 0.56. The effect is large (Cohen's *d* = 5.94) and the difference is significant at *p* < 0.01 (cf. Figure 4).

This was reflected in the examination of the pressure pain of the different muscle groups. The patients were characterized by being significantly more affected in all examined muscles except the left paravertebral muscles of the lumbar spine compared to the healthy control group. A large effect could be identified in the left and right M. sternocleidomastoideus (Cohens’ *d* = 1.10/0.92), left and right M. scaleni (Cohens’ *d* = 1.15/1.02), left M. trapezius (Cohens’ *d* = 0.98), left and right M. levator scapulae (Cohens’ *d* = 1.34/1.60), left and right M. rhomboideus (Cohens’ *d* = 1.62/1.26), left and right paravertebral muscles of the cervical spine (Cohens’ *d* = 1.01/1.01) and the left M. quadratus lumborum (Cohens’ *d* = 0.81).

There was a significant difference in the pressure pain of the temporomandibular joint with a small effect (Cohens’ *d* = 0.45) as well as the joint muscles with a medium effect (Cohens’ *d* = 0.67) could be described.

As shown in Table 4, matching the results of the pressure pain evaluation, a significant difference in most of the examined muscles regarding the muscle tension of the patients before therapy compared to the healthy control group was found. Especially the difference between the two groups in the muscle tension of M. rhomboideus was noticeable. On average, 88% of the patients had high muscle tension in the right M. rhomboideus (vs. 84% in the left M. rhomboideus) in contrast to only 31% (vs. 34% in the left) of the healthy participants (*p* = 0.0001), both sides showing a large effect size (Cohens’ *d* = 1.38 vs. 1.16).

**Table 4:** Comparison of the muscle tension of examined muscles: patient group (before therapy) vs. healthy control group.

	Patients in T1		Control Group		*p*-Value
	*N*	Mean ± sd	*N*	Mean ± sd	
Hyoid muscles right	32	0.34 ± 0.48	32	0.06 ± 0.25	0.006
Hyoid muscles left	32	0.31 ± 0.47	32	0.06 ± 0.25	0.011
M. sternocleidomastoideus right	32	0.47 ± 0.51	32	0.28 ± 0.46	0.012
M. sternocleidomastoideus left	32	0.53 ± 0.51	32	0.22 ± 0.42	0.010
M. scaleni right	32	0.91 ± 0.30	32	0.56 ± 0.50	0.002
M. scaleni left	32	0.91 ± 0.30	32	0.59 ± 0.50	0.004
M. trapezius right	32	0.94 ± 0.25	32	0.97 ± 0.18	0.557
M. trapezius left	32	0.94 ± 0.25	32	0.94 ± 0.25	1.000
M. levator scapulae right	32	0.93 ± 0.25	32	0.75 ± 0.44	0.040
M. levator scapulae left	32	0.91 ± 0.30	32	0.78 ± 0.42	0.171
M. rhomboideus right	32	0.88 ± 0.34	32	0.31 ± 0.47	0.000
M. rhomboideus left	32	0.84 ± 0.37	32	0.34 ± 0.48	0.000
M. pectoralis right	32	0.81 ± 0.40	31	0.61 ± 0.50	0.082
M. pectoralis left	32	0.81 ± 0.40	31	0.52 ± 0.51	0.013
Paravertebral muscles cervical spine right	32	0.38 ± 0.49	32	0.13 ± 0.34	0.022
Paravertebral muscles cervical spine left	32	0.38 ± 0.49	32	0.13 ± 0.34	0.022
Paravertebral muscles thoracic spine right	32	0.47 ± 0.51	32	0.28 ± 0.46	0.124
Paravertebral muscles thoracic spine left	32	0.47 ± 0.51	32	0.25 ± 0.44	0.070
Paravertebral muscles lumbar spine right	32	0.53 ± 0.51	32	0.47 ± 0.51	0.624
Paravertebral muscles lumbar spine left	32	0.50 ± 0.51	32	0.47 ± 0.51	0.806
M. quadratus lumborum right	32	0.88 ± 0.34	32	0.59 ± 0.50	0.012
M. quadratus lumborum left	32	0.88 ± 0.34	32	0.63 ± 0.49	0.021

All variables are median values ± standard deviation.

The widespread pain score showed a significant difference between the groups (*p* = 0.0001). Whereas the patient group scored three times as high as the healthy subjects with 9.4, the healthy control only scored around 3 points (large effect size, Cohens’ *d* = 1.74).

Since temporomandibular disorders are known to be associated with playing an instrument (30), we additionally evaluated the existence of temporomandibular dysfunctions in our study participants. Thereby, no significant differences between the two groups were found. Regarding the Beighton score for detecting the association of PRMD with hypermobility, the healthy group scored on average 2.78 points. In comparison, the patients scored 3.43 points, so both groups were diagnosed with moderate hypermobility. Even though it was not significant, there is a difference in those being diagnosed with generalized hypermobility by reaching a Beighton score of five or even more. In the patient group, 12 of the 32 (37,5%) probands were diagnosed with generalized hypermobility, whereas in the control group, 8 of the 32 (25%) students did reach a Beighton score of five or even higher.

Consistent with the result of existing moderate hypermobility in both groups, we provide a higher mobility of the cervical spine in both groups compared to the normal range of inclination of 45° (31). The mean inclination of the patients in T1 was 66.5°, whereas the healthy subjects were able to incline their cervical spine on average to 72.81°. A difference of 6,31° between the two groups was found to be significant (*p* < 0.0232) with a medium effect size (Cohens’ *d* = 0,58).

Since both groups were diagnosed with moderate hypermobility, as expected, no relevant differences could be detected by measuring the finger to floor distance between the patients and the control group.

### Patients in T1 vs. patients in T2

For estimating the effectiveness of the physiotherapeutic therapy, a second examination of the patient group was carried out after the treatment.

The VAS1 of the musicians suffering from PRMD declined significantly from the initial 5.3 (T1) on average to 3.4 (T2) (*p* < 0.0001) and the effect was large (Cohens’ *d* = 1.42). The maximum pain was declared at 8.2 in T1, and 7.2 in T2.

**Figure 5:**
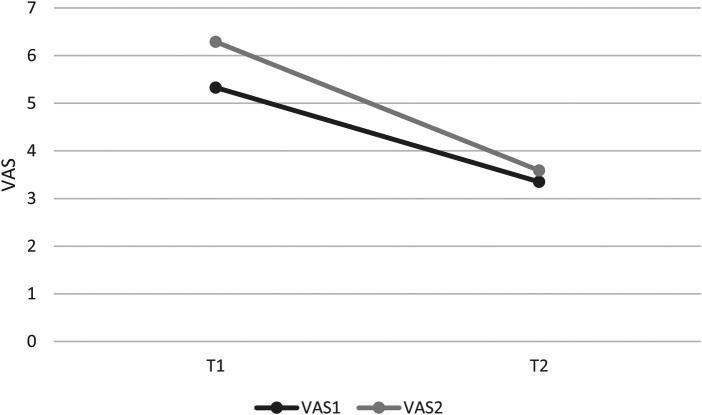
VAS before (T1) and after (T2) therapy.

The VAS2 was significantly reduced (*p* < 0.0001). In T1, the average VAS2 was 6.28, in T2 the VAS2 was measured at 3.58 on average, which means a reduction of 57%, after twelve sessions of therapy, which resembles a large effect (Cohens’ *d* = 1.19). Figure 5 shows VAS1 before and after therapy for the patient group. VAS2 is higher than VAS1 before and after therapy. Both decrease significantly after therapy.

The detailed examination of the pressure pain in T2 showed a significant reduction, with an average of 33% of patients having less pressure pain in the M. levator scapulae on both sides than before the therapy (*p* < 0.0116), showing a medium effect (Cohens’ *d* = 0.66). The pressure pain of the M. rhomboideus was reduced from 69% in T1 to 38% on average in T2 on the right side, and from 72% (T1) to 44% (T2) on the left side (medium effect, Cohens’ *d* = 0.65/0.58). Interestingly, the pressure pain examination of the M. sternocleidomastoideus only showed a significant reduction of the left side (T1: 59%; T2: 38%) (medium effect, Cohens’ *d* = 0.59). The pressure pain of the paravertebral muscles of the cervical spine was also only significantly reduced on the right side (T1: 34%; T2: 13%)(medium effect, Cohens’ *d* = 0.53). Table 5 shows the differences in the pressure pain of the examined muscles before and after therapy and in comparison to the CG.

**Table 5:** Effect of the manual therapy on the pressure pain of the patient’s muscles.

	Patients in T1		Patients in T2		Control Group		*p*-Value
	*N*	Mean ± sd	*N*	Mean ± sd	*N*	Mean ± sd	
Hyoid muscles right	32	0.38 ± 0.49	32	0.16 ± 0.37	32	0.03 ± 0.18	0.040
Hyoid muscles left	32	0.28 ± 0.46	32	0.13 ± 0.34	32	0.03 ± 0.18	0.123
M. sternocleidomastoideus right	32	0.56 ± 0.50	32	0.47 ± 0.51	32	0.16 ± 0.37	0.461
M. sternocleidomastoideus left	32	0.59 ± 0.50	32	0.38 ± 0.49	32	0.13 ± 0.34	0.082
M. scaleni right	32	0.88 ± 0.34	32	0.72 ± 0.46	32	0.44 ± 0.50	0.123
M. scaleni left	32	0.81 ± 0.40	32	0.71 ± 0.46	32	0.31 ± 0.47	0.384
M. trapezius right	32	0.88 ± 0.34	32	0.78 ± 0.42	32	0.56 ± 0.50	0.324
M. trapezius left	32	0.91 ± 0.30	32	0.81 ± 0.40	32	0.50 ± 0.51	0.285
M. levator scapulae right	32	0.75 ± 0.44	32	0.44 ± 0.50	32	0.13 ± 0.34	0.012
M. levator scapulae left	32	0.75 ± 0.44	32	0.44 ± 0.50	32	0.19 ± 0.40	0.395
M. rhomboideus right	32	0.69 ± 0.47	32	0.38 ± 0.49	32	0.16 ± 0.37	0.012
M. rhomboideus left	32	0.72 ± 0.46	32	0.44 ± 0.50	32	0.09 ± 0.30	0.024
M. pectoralis right	32	0.84 ± 0.37	32	0.84 ± 0.37	31	0.52 ± 0.51	1.000
M. pectoralis left	32	0.78 ± 0.42	32	0.78 ± 0.42	31	0.48 ± 0.51	1.000
Paravertebral muscles cervical spine right	32	0.34 ± 0.48	32	0.13 ± 0.34	32	0.0 ± 0.0	0.040
Paravertebral muscles cervical spine left	32	0.34 ± 0.48	32	0.19 ± 0.40	32	0.0 ± 0.0	0.160
Paravertebral muscles thoracic spine right	32	0.28 ± 0.46	32	0.22 ± 0.42	32	0.06 ± 0.25	0.567
Paravertebral muscles thoracic spine left	32	0.38 ± 0.49	32	0.22 ± 0.42	32	0.09 ± 0.30	0.175
Paravertebral muscles lumbar spine right	32	0.28 ± 0.46	32	0.22 ± 0.42	32	0.09 ± 0.30	0.567
Paravertebral muscles lumbar spine left	32	0.22 ± 0.42	32	0.19 ± 0.40	32	0.09 ± 0.30	0.758
M. quadratus lumborum right	32	0.72 ± 0.46	32	0.53 ± 0.51	32	0.89 ± 0.49	0.124
M. quadratus lumborum left	32	0.78 ± 0.42	32	0.66 ± 0.48	32	0.40 ± 0.50	0.270

All variables are median values ± standard deviation (sd).

Concerning the M. quadratus lumborum, no significant reduction of the pressure pain was achieved, but concerning hypertension, a significant decrease was only seen on the right side with a medium effect (Cohens’ *d* = 0.66) proven. On average, 73% of the patients had hypertension of the muscle, whereas in T2 only 59,3% of patients on average were still suffering from hypertension. No other significant reduction of hypertensive muscles could be detected.

**Figure 6:**
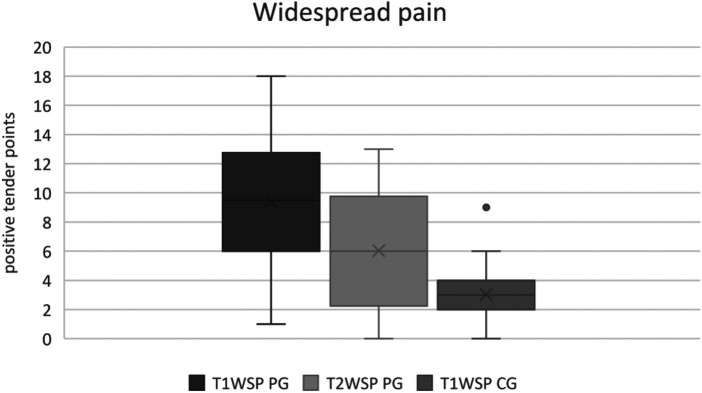
Widespread pain.

Matching the significantly decreased VAS after therapy, a significant reduction of the WSP was also achieved. The effect was medium (Cohens’ *d* = 0.78) and the difference was significant at *p* < 0.0005. On average, the patients had 9.4 positive tender points in T1 (T1WSPP), and 28% scored a positive WSP-testing since they had twelve or more positive tender points. The percentage of the positively tested WSP patients in T2 decreased to 9%. In comparison, the healthy probands had on average, 3 positive tender points, and none of them scored a positive WSP-testing (cf. Figure 6).

## Discussion

The most important result showed that a significant decrease in pain could be achieved, from the initial average of 5.3 on the VAS before therapy to 3.4 afterward, equaling an improvement of pain level by 64%. Since the therapy was limited to 12 sessions per patient, an even higher reduction through the continuation of the therapy seems likely. Moreover, additional forms of therapy might help to reduce the symptoms as found by Sousa et al. (32), who showed the positive effect of self-administered exercises based on Tuina techniques on musculoskeletal disorders of professional orchestra musicians. Tuina is part of Chinese manual therapy, using different techniques consisting of vibration, pressure, warming, and movement. The use of a practice diary, additional strengthening, and movement or flexibility regimes (off the instrument), as well as muscle activation patterns specific to the instrument (19) are also recommended. Furthermore, it is recommended that patients perform physical activities regularly, as they are an important element in the prevention of musculoskeletal disorders among young musicians (33).

As demonstrated by Zaza and Farewell (18), pausing the instrument decreases PRMD. However, this was not a realistic measure in our study, since participating music students were not able to interrupt their studies. The assumption that a more intense, longer, and even more specific treatment might improve the outcome further. Therefore, more studies with a longer period of physiotherapeutic treatment and a multimodal therapy concept should be designed.

This study provides evidence of a correlation between the VAS and the pressure pain of the different muscles. Whereas 13.1 of the muscles in the patient group were determined on average as painful when examined, only 4.9 of the healthy group muscles on average offered a painful examination.

In keeping with the reduction of the pain level, a significant reduction of pressure pain of the M. rhomboideus and the M. levator scapulae could also be achieved by therapy on the right, as well as on the left side. It is noteworthy that both muscles are important for the stabilization and movements of the scapula, and that they frequently show painful trigger points.

Since those muscles play an important role in a lot of movements to enable musicians to play their instruments, as well as stabilize the body posture while playing, they were shown to often be painful in examination compared to the healthy control group, and a more effective therapeutic intervention could be designed, focusing on relaxation and tension management of rhomboideus and levator scapulae muscles. To prevent muscle dysfunctions in those muscles in musicians, another preventing factor is practicing a good body posture while playing their instrument, for example by implementing Alexander Technique (34).

Interestingly, in our study, only the pressure pain of the right paravertebral cervical spine muscles, and only the left side of the M. sternocleidomastoideus significantly decreased. A possible cause could be that the physiotherapist concentrated on the most painful muscles, which might be required more than the others. It is known that high string players in particular, have an asymmetric muscle load while playing their instrument and therefore suffer more often from musculoskeletal pain (35, 36). Since our study did not subdivide the patients into subgroups of instrument played, this could explain why not all of the initially painful muscles had a significant decrease in pain after the therapy. Further studies are needed to determine if a longer period of therapy with a standardized, and more specific therapy regimen for each instrument affects all of the painful muscles.

Our study detected important differences between healthy music students and those suffering from PRMD. For example, the sideward inclination of the head of the control group was significantly higher. This might correlate with the patient groups having higher muscle tension in the paravertebral cervical spine muscles compared to the healthy group, meaning the movement of the head could be limited by them being tense and therefore shortening cervical spine muscles.

On average the Beighton score did not differ in the two groups, but the percentage of those being diagnosed with generalized hypermobility was 37.5% higher in the patient group than in the control group. Here, only 25% were diagnosed with generalized hypermobility. The percentage of those being diagnosed with generalized hypermobility is noticeable in both groups compared to a university-aged population. Here 14.2% out of the 654 participants had a Beighton score of five or more (37). This leads to the assumption, that musicians in general might benefit from a higher mobility of their joints, as is often described concerning the exceptional musician Nicolò Paganini (38). As already suggested, “the musculoskeletal symptoms associated with practice and performance [of musicians] may be due to a lack of hypermobility of some joints involved in repetitive motion, or due to hypermobility of joints not involved in repetitive motion but associated with support function” (39). It would be interesting to examine if there is a direct correlation between hypermobility of the joints required for playing a specific instrument and hypertension of the stabilizing muscle. Therefore, to detect if hypermobility is also associated with a higher risk of developing PRMD, further studies are needed.

It is also significant that the two groups, as well as T1 vs. T2, differ regarding the WSP. This tool was used since the WSP is an established method of quantifying the felt pain in patients. Our results suggest that the patient's group WSP could be significantly reduced through physiotherapeutic treatment.

As WSP could be interpreted as a risk factor for developing chronic pain one could speculate that an early intervention against PRMD could also lower the risk of chronic course PRMD.

### Strengths of the study

For the first time, this study shows evidence of the positive effect of manual therapy on PRMD. Data collection was carried out by a detailed clinical examination of every participant with a focus on especially small muscle groups that have not been described to date in other studies. To achieve accurate and objective data, we used specialized equipment (e.g., CROM, InBody Machine), and the probands were examined by two experienced examiners. We documented a correlation to other often with musician associated syndromes, such as hypermobility. Furthermore, our sample, though relatively small, was also characterized by an excellent matching of the control group by age and gender.

The patients received therapy from highly qualified physiotherapists with long-term experience in the therapy of musicians and PRMD.

### Limitations of the study

The main weakness of the study is the case-control follow-up design, documenting improvements due to manual therapy. However, this could also be the effect of spontaneous recovery. We would like to emphasize that during ongoing music studies and under the stress of the approaching exams at the end of the semester, PRMDs usually increase and do not improve spontaneously. Indeed, we first planned a prospective randomized trial, applying the intervention and a sham intervention in PRMD students. However, due to the high level of suffering and the high pressure to be able to perform for those being concerned, we decided not to implement a control group. Since the study participants were music students at the beginning of their careers, we did not want to risk a chronification of prolonged pain and the distress this might cause. To be able to detect the influence of the placebo effect more studies are needed, which might examine a different group of study participants.

Since the study does not distinguish between the different instruments, which often need a typical posture, more specific muscular patterns for each instrument should be investigated. This may help to develop standardized therapy concepts to avoid chronic pain as a result of the instrument played. Because of the difficulties in time management experienced by music students, the therapy was not always carried out over six weeks, twice per week, as recommended. A potentially stronger effect might be achieved through stricter timing of the therapies. Furthermore, we might further discuss how musicians tend to rate their health more positively (7), and further studies with a control group of non-musicians might be helpful. As mentioned above, due to ethical concerns we did not include a placebo group.

### Conclusion

When PRMDs are detected at an early stage, physiotherapeutic treatments provide the opportunity to reduce pain significantly. A reduction in muscle tension can additionally be achieved by therapy, which can help to regain the normal range of motion that is required to play the instrument. For developing specific therapies, more studies are needed, including different kinds of instrumentalists and an enlarging of the study population. More controlled trials are needed to find the most effective evidence-based therapy to keep those making music for others physically healthy, and contributing to the well-being of people who experience their music.

## Data Availability

The raw data supporting the conclusions of this article will be made available by the authors, without undue reservation.
