# Corrigendum: Effect of manual therapy on music students with playing-related musculoskeletal disorders: a prospective study

**DOI:** 10.3389/fpain.2023.1303660

**Published:** 2023-10-12

**Authors:** Carolin Assel

**Affiliations:** Hannover Medical School, Hanover, Germany

**Keywords:** playing-related musculoskeletal disorder, manual therapy, hypermobility, music students, musician, pain, music

A Corrigendum on Effect of manual therapy on music students with playing-related musculoskeletal disorders: a prospective study By Assel C, Nugraha B, Kallusky N, Faßnacht-Lenz S, Altenmüller E, Gutenbrunner C, et al. (2023) Front. Pain Res. 4:1151886. doi: 10.3389/fpain.2023.1151886

## Text correction

In the published article, there was an error. Incorrect numerical information was given in the Abstract concerning the VAS.

A correction has been made to Abstract. This sentence previously stated:

“After analyzing the data of the patient group (PG) a significant reduction of pain level on the VAS from an average pain of 6.31 to 3.53 was found (large effect).”

The corrected sentence appears below:

“After analyzing the data of the patient group (PG) a significant reduction of pain level on the VAS from an average pain of 5.33 to 3.35 was found (large effect).”

The authors apologize for this error and state that this does not change the scientific conclusions of the article in any way. The original article has been updated.

